# Supplementary figures and images for: Early detection of abiotic stress in plants through SNARE proteins using hybrid feature fusion model
Source: PeerJ Comput Sci. 2024 Aug 5;10:e2149. doi: 10.7717/peerj-cs.2149 (PMC11323173; doi:10.7717/peerj-cs.2149)

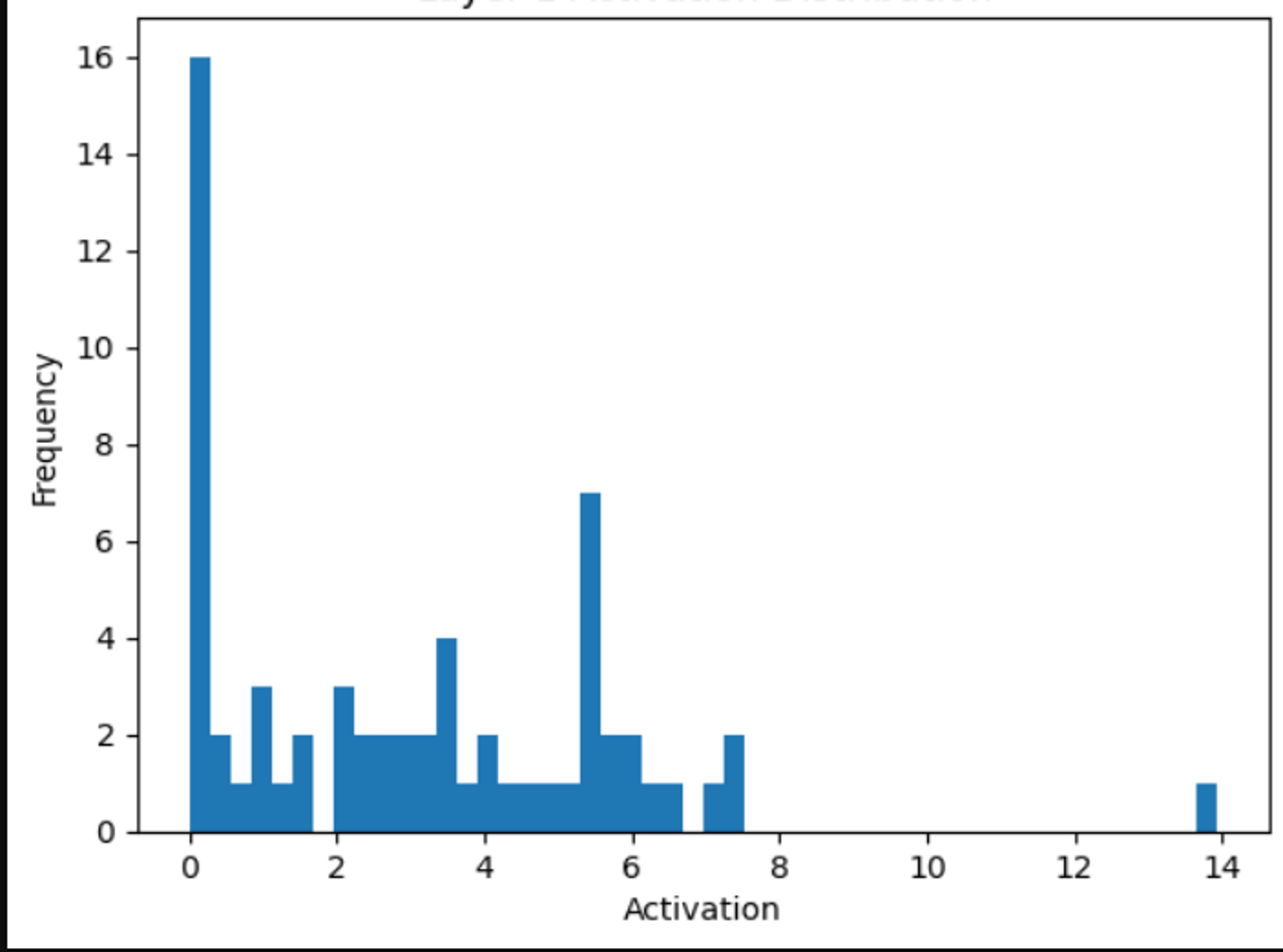

Supplement: Supplemental Information 2 [file peerj-cs-10-2149-s002.png]

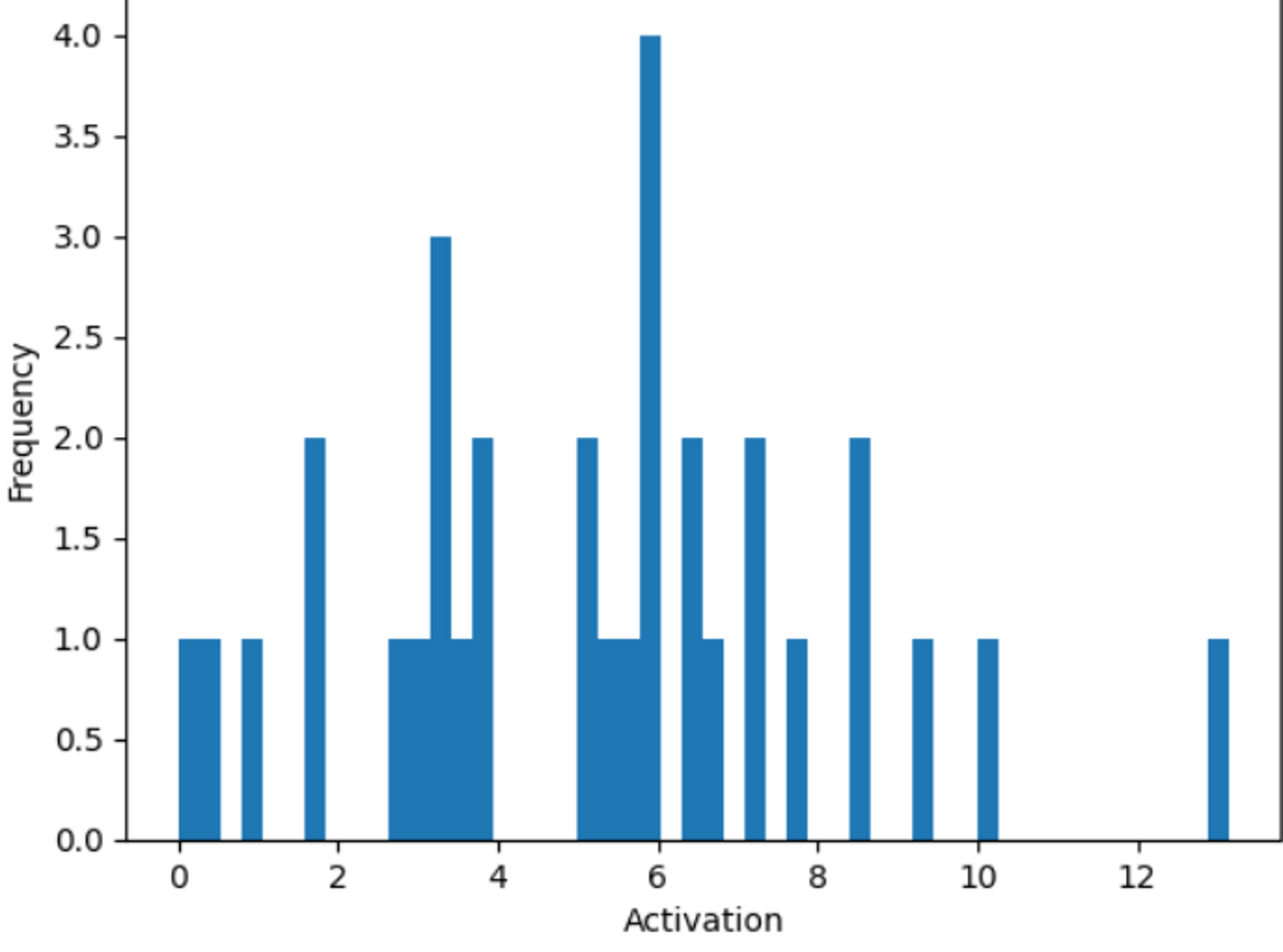

Supplement: Supplemental Information 3 [file peerj-cs-10-2149-s003.png]

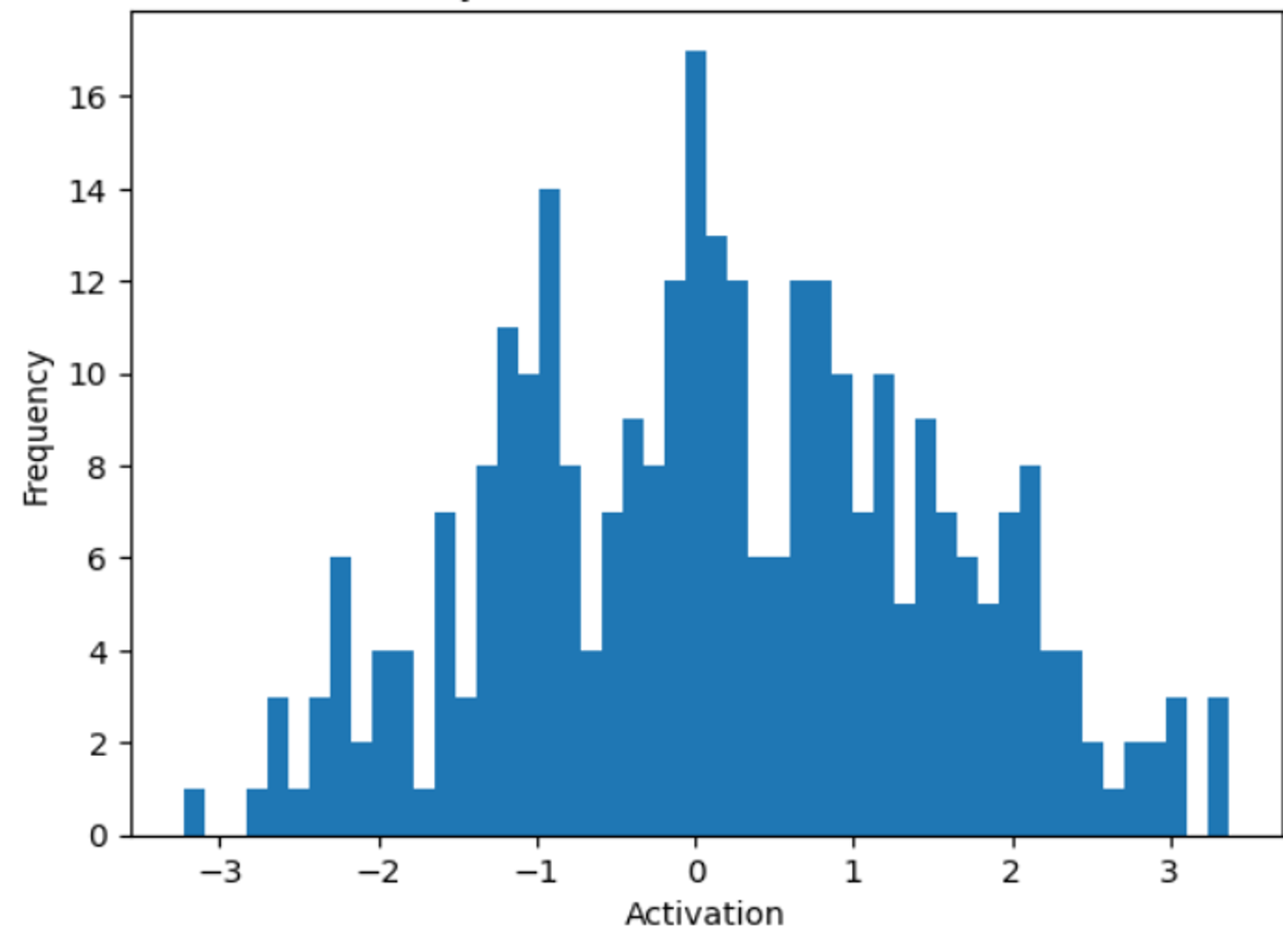

Supplement: Supplemental Information 4 [file peerj-cs-10-2149-s004.png]

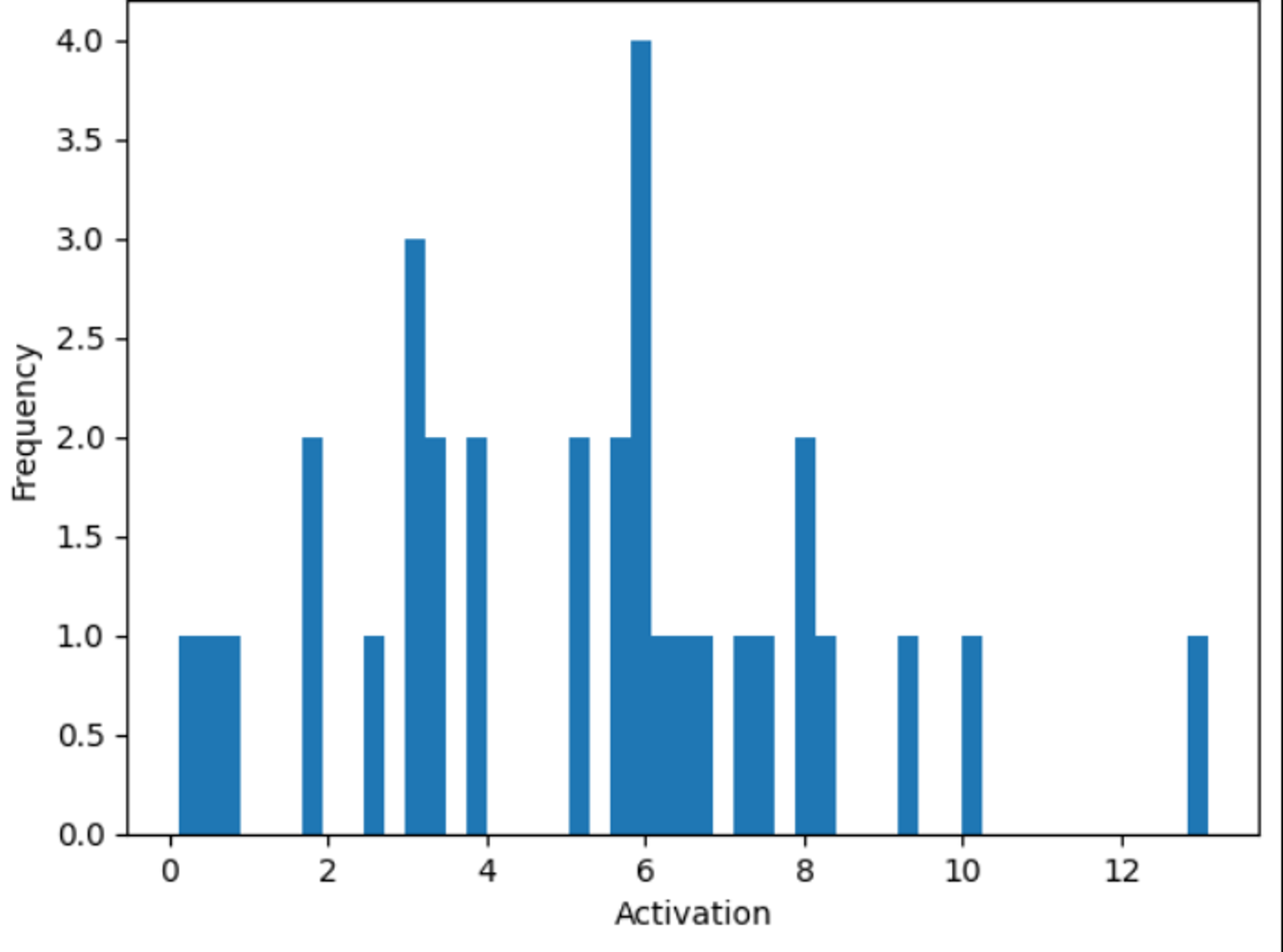

Supplement: Supplemental Information 5 [file peerj-cs-10-2149-s005.png]

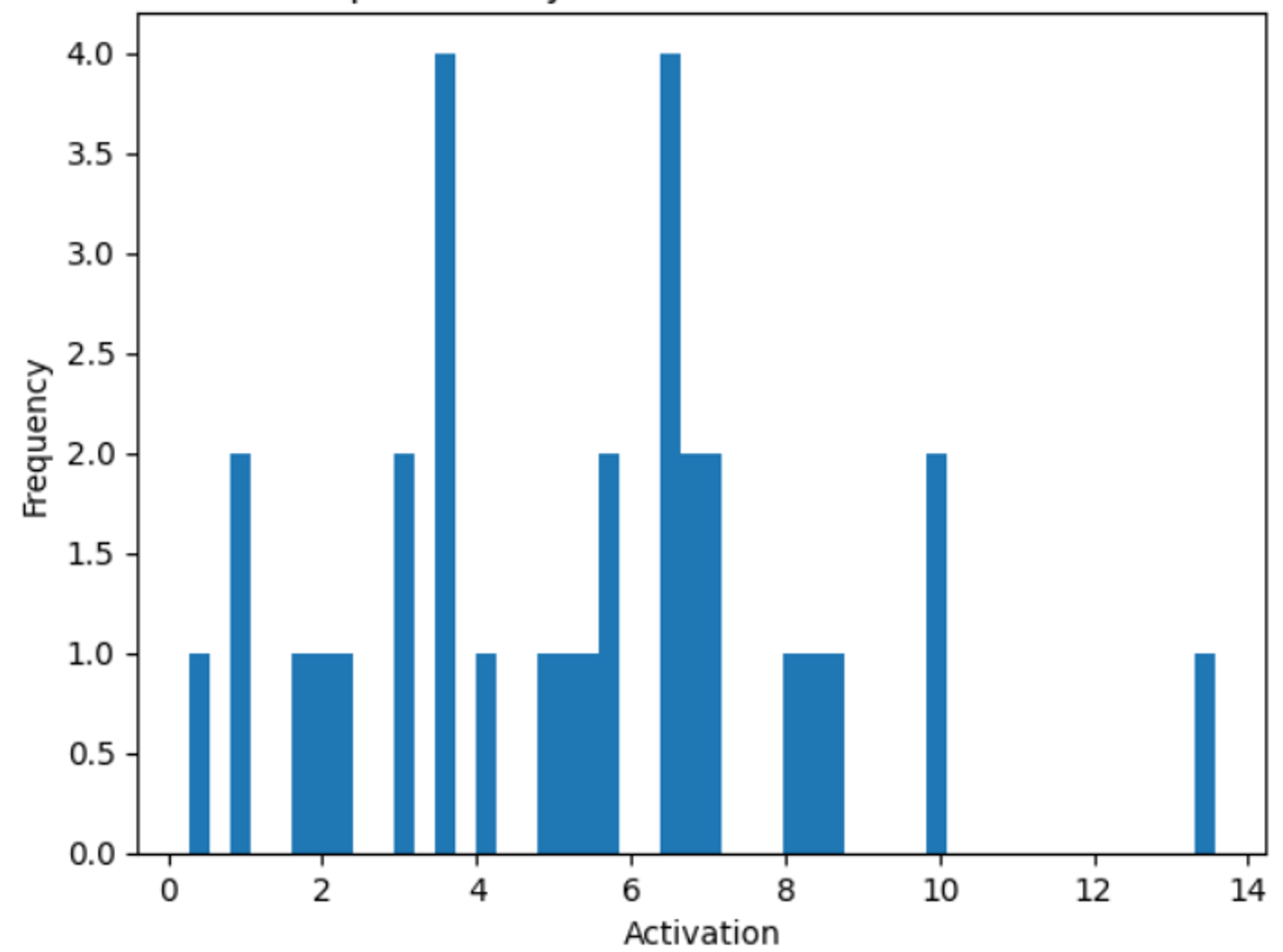

Supplement: Supplemental Information 6 [file peerj-cs-10-2149-s006.png]

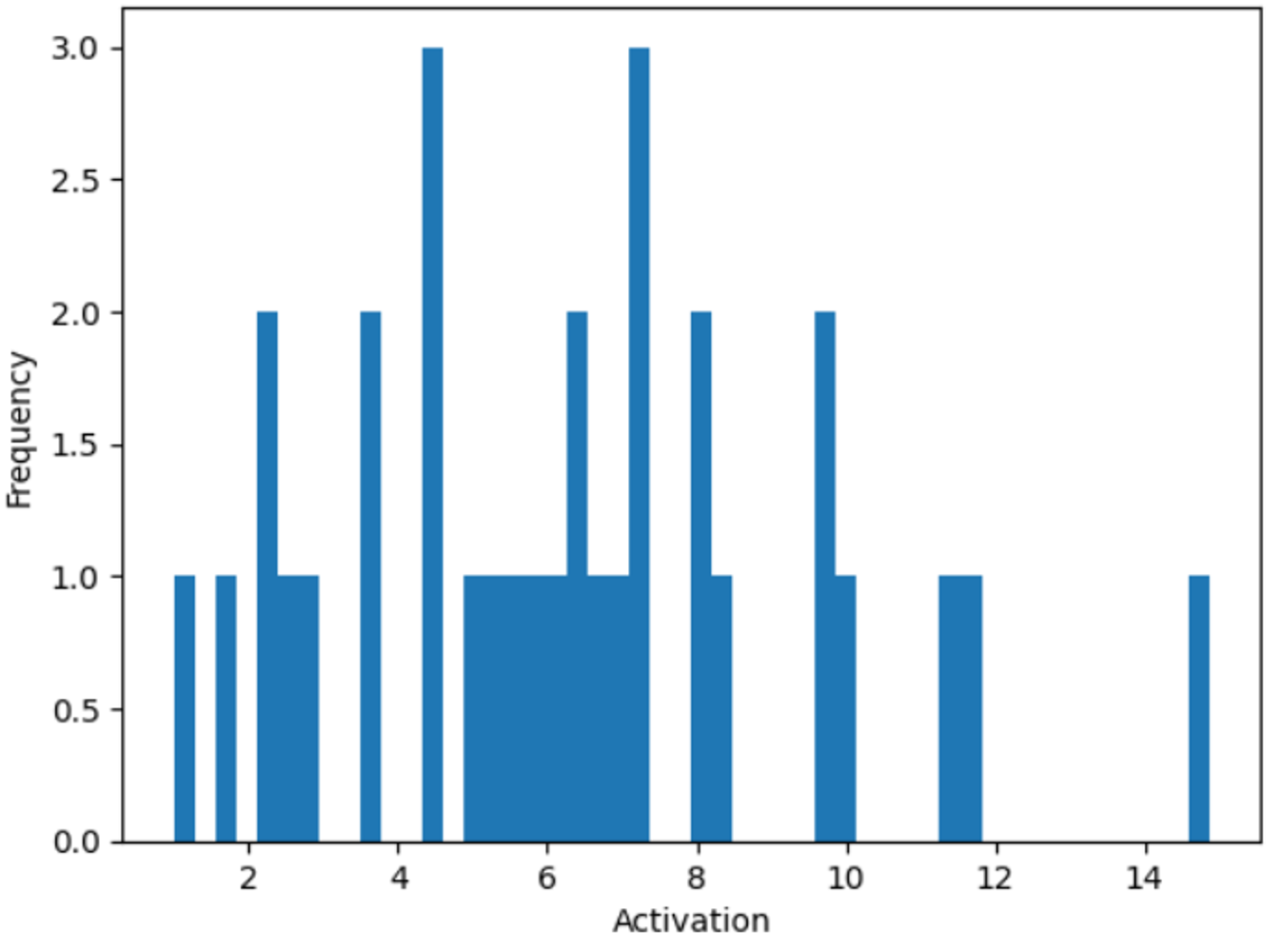

Supplement: Supplemental Information 7 [file peerj-cs-10-2149-s007.png]

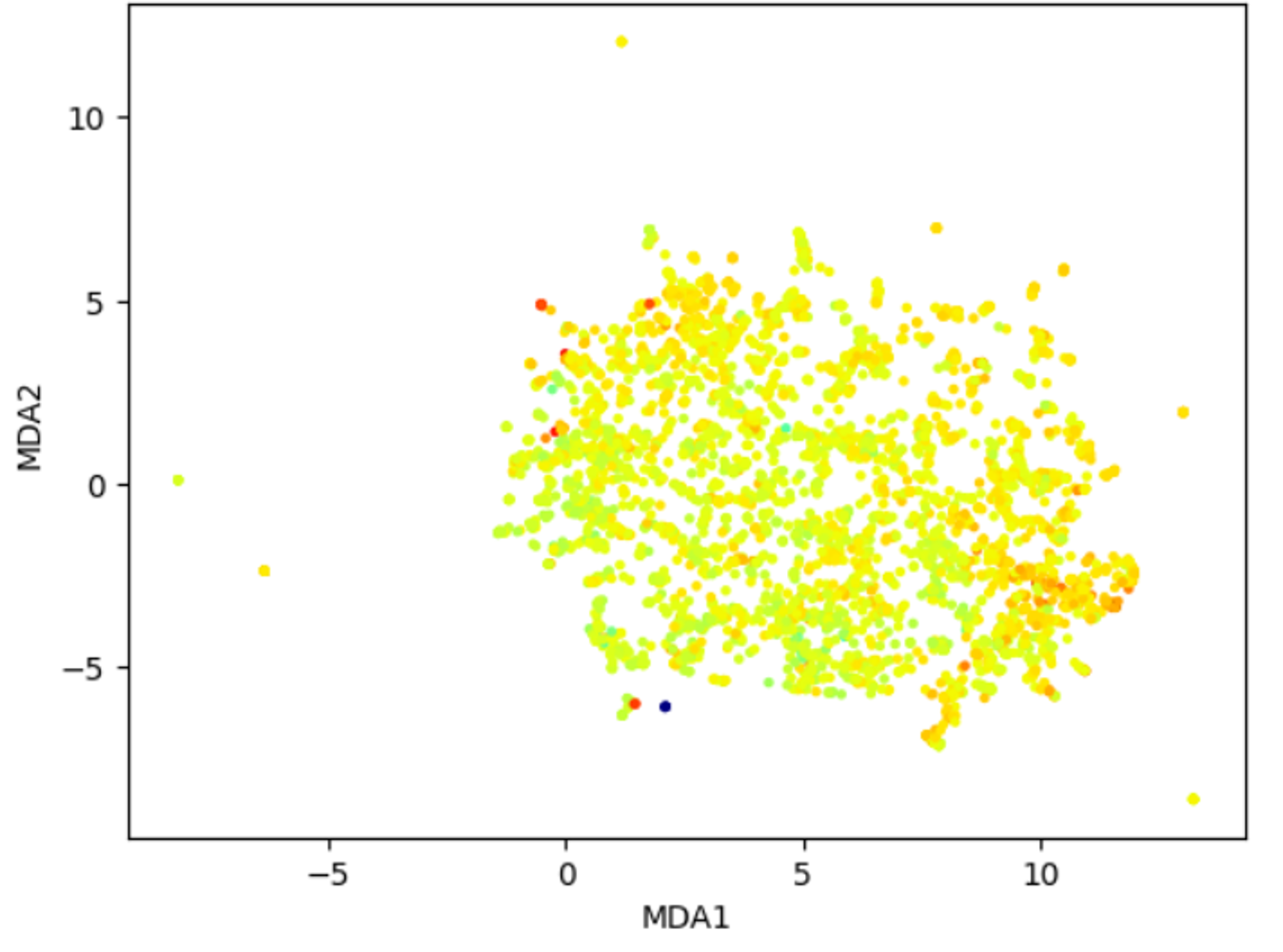

Supplement: Supplemental Information 8 [file peerj-cs-10-2149-s008.png]

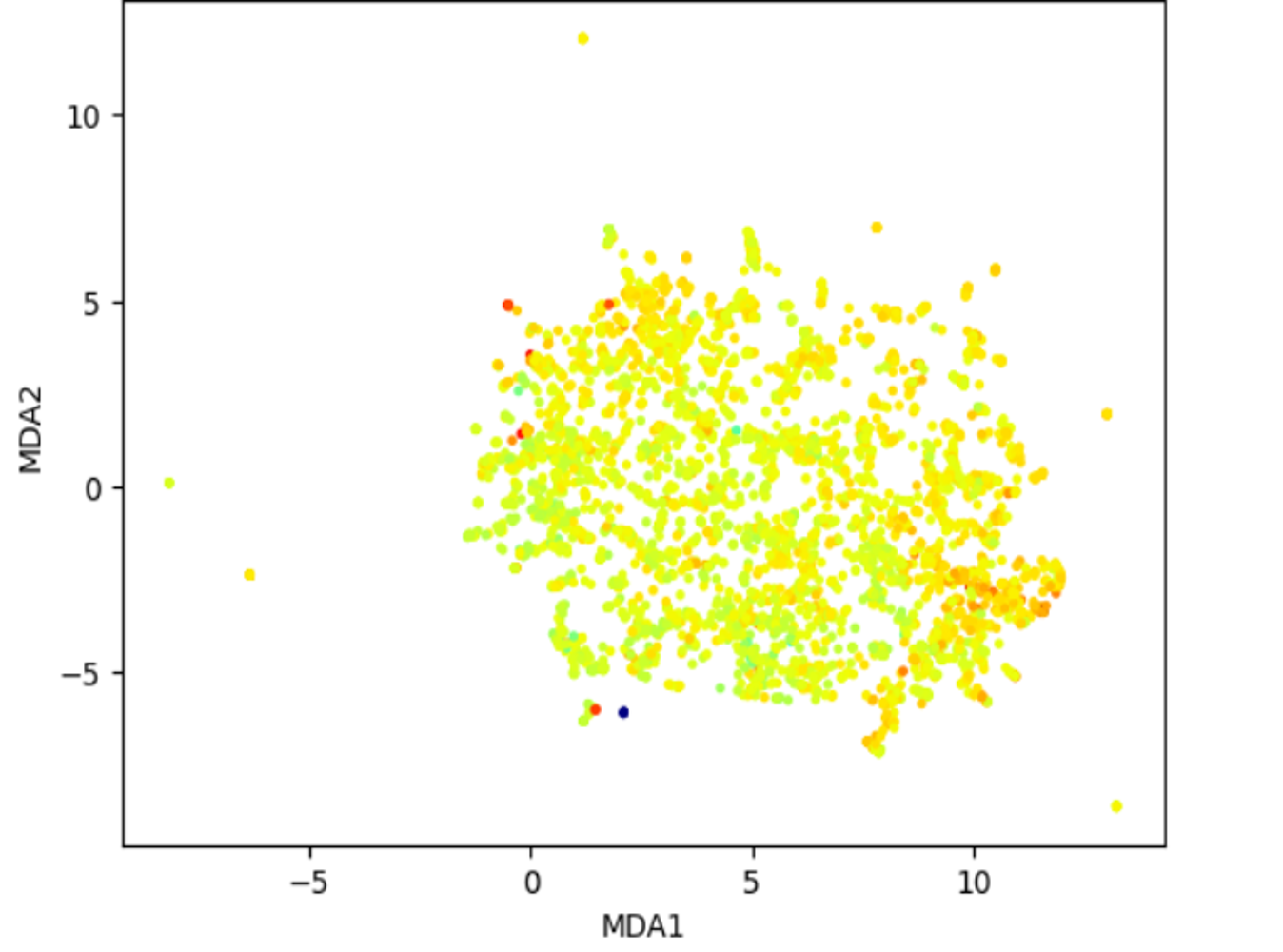

Supplement: Supplemental Information 9 [file peerj-cs-10-2149-s009.png]

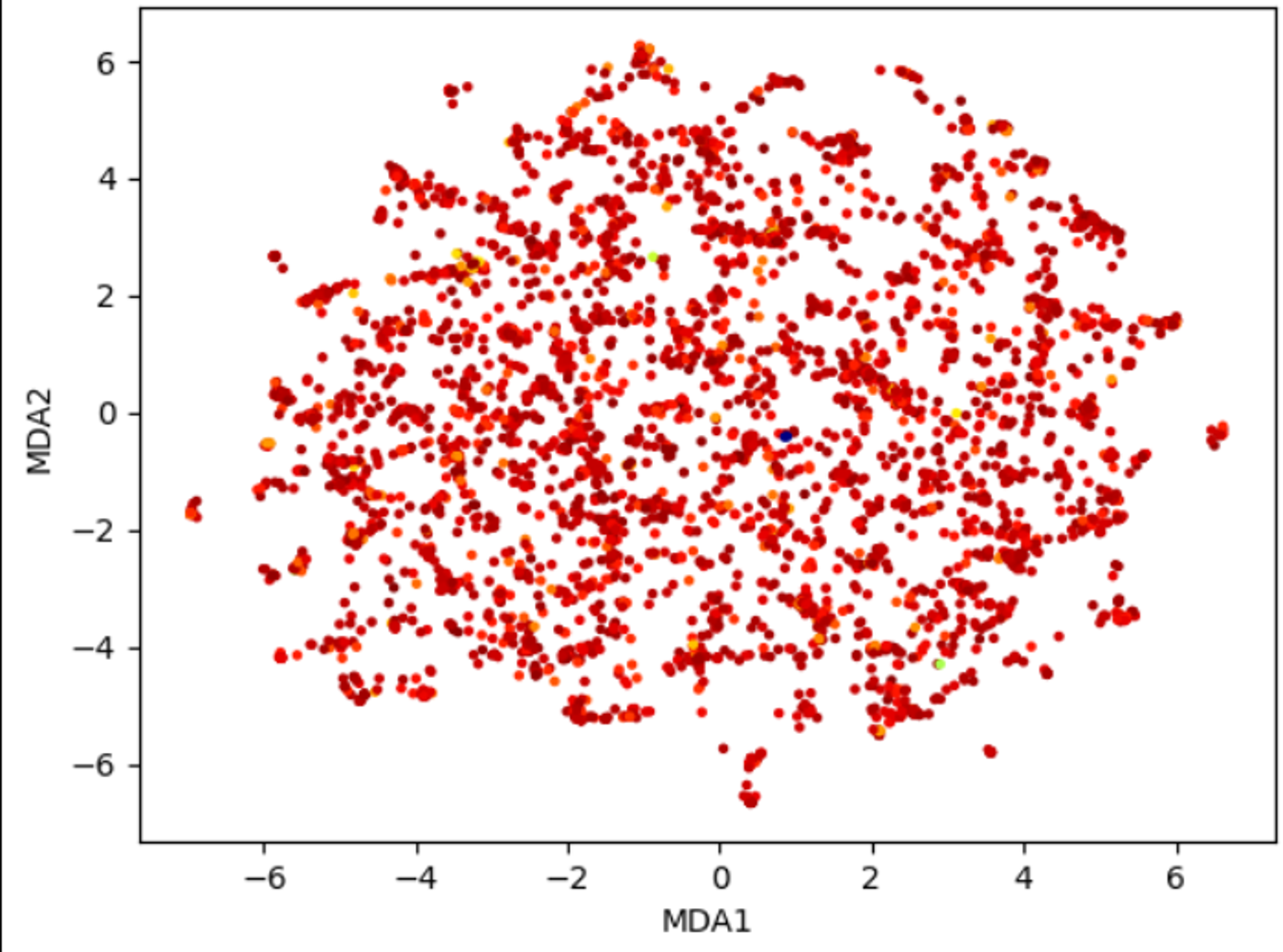

Supplement: Supplemental Information 10 [file peerj-cs-10-2149-s010.png]

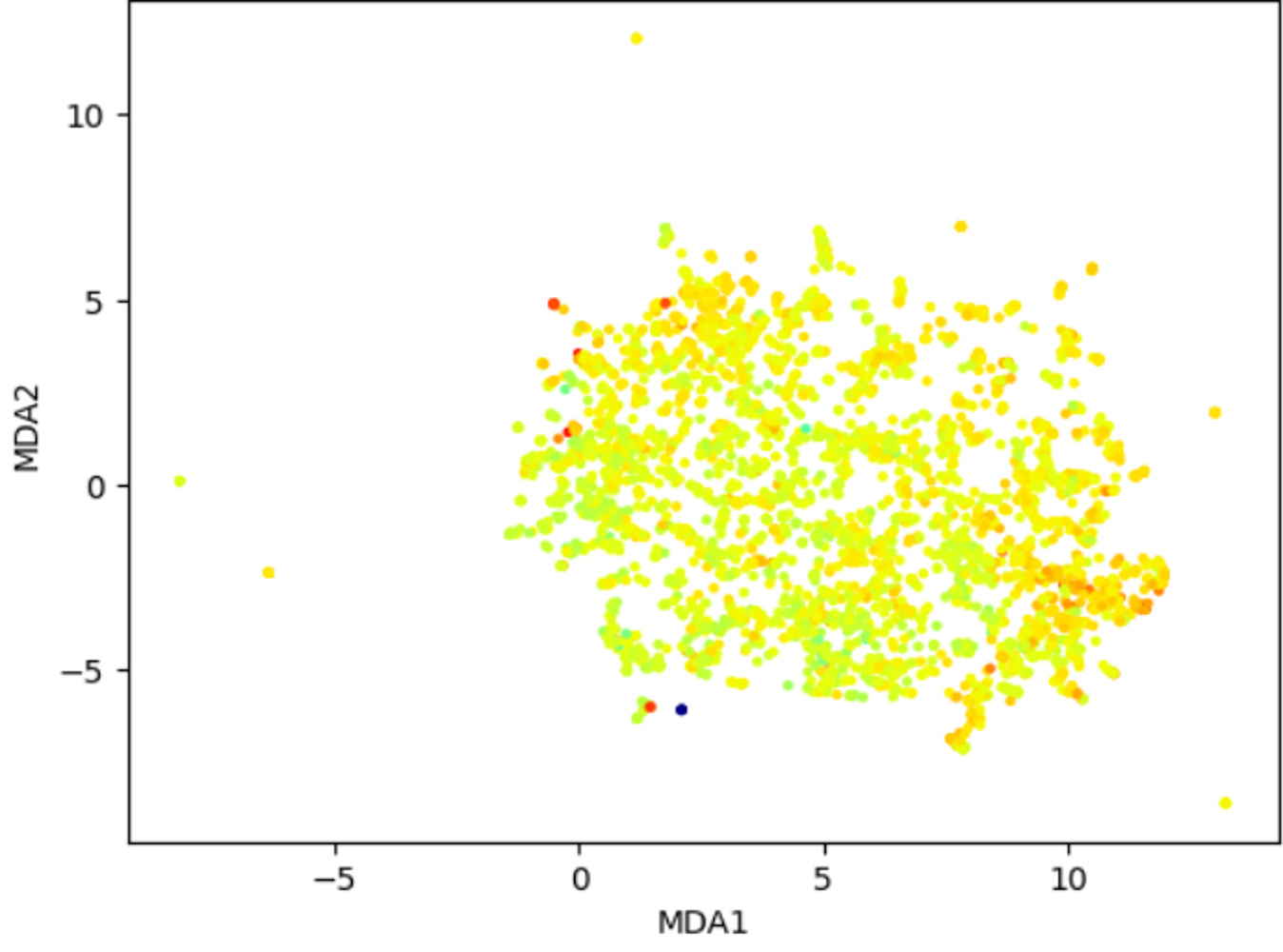

Supplement: Supplemental Information 11 [file peerj-cs-10-2149-s011.png]
